# Total Correction for Tetralogy of Fallot in Patients Weighing Over 10 kg: Experiences and Follow-Up Outcomes

**DOI:** 10.7759/cureus.63133

**Published:** 2024-06-25

**Authors:** Poorna Chandhar, Subrata Pramanik, Anubhav Gupta, Manju Gupta

**Affiliations:** 1 Department of Cardiac/Thoracic/Vascular Surgery, Vardhman Mahavir Medical College and Safdarjung Hospital, New Delhi, IND

**Keywords:** tetralogy of fallot (tof), above 10 kg total correction, transannular patch (tap), adolescence and adulthood tof, non-ventriculotomy infundibular resection, rvot patch augmentation

## Abstract

Background

Although the recommended time for total correction of tetralogy of Fallot (TOF) is during infancy, sometimes TOF cases present to healthcare setups after pre-school age, with some cases presenting even beyond adolescence in developing countries. The objective of this study was to assess patients with TOF weighing 10 kg and above who underwent definitive corrective surgical techniques such as transannular patch (TAP), valve-sparing right ventricular outflow tract (RVOT) pericardial patch augmentation, non-ventriculotomy infundibular resection for postoperative complications, hospital stay, and right ventricular (RV) dysfunction in the immediate postoperative period and subsequent outpatient department follow-ups.

Methodology

This comprehensive, retrospective cohort study included single-center data collected between January 16, 2018, and January 15, 2024. The study included 63 patients diagnosed with TOF weighing 10 kg and above, ensuring a robust and representative sample.

Results

Of the 119 patients who underwent total correction for TOF, 63 met the study’s inclusion criteria of TOF weighing above 10 kg. Of the 63 patients, 55.6% were males, and 44.4% were females. The mean weight of the study participants was 33.4 kg. The mean age was 15.9 years. Of the 63 patients, 39 underwent TAP surgery, 18 underwent RVOT patch augmentation, and six underwent total correction by non-ventriculotomy infundibular resection. There was a significant difference between the type of surgery groups and RV dysfunction, with the TAP group showing a higher incidence of RV dysfunction, indicating a potential risk factor associated with this technique.

Conclusions

Although TAP has significant immediate postoperative complications compared to other techniques, its long-term follow-up suggests that long-term survival and quality of life, as measured by major adverse cardiac events such as heart failure, arrhythmias, and reoperation rates, are commensurable in adulthood. This indicates that despite the initial challenges, TAP can provide satisfactory outcomes in the long run.

## Introduction

About 7-10% of all congenital cardiac defects are caused by the most common cyanotic congenital heart disease (CCHD) known as the tetralogy of Fallot (TOF) [1,2], occurring in 4 out of 10,000 live babies. Pulmonary artery stenosis (PS), ventricular septal defect (VSD), right ventricular hypertrophy (RVH), and overriding of the aorta, which is caused by anterior and superior deviation of the infundibular septum, are the four fundamental structural defects that comprise this condition [3]. In addition, atrial septal defect (ASD) and TOF comprise the pentalogy of Fallot.

In developed countries, TOF is typically diagnosed and corrective surgery is attempted at a younger age [4]. The first year of life is the ideal period for total correction, as suggested by established literature such as the study by Starr et al., which proposed six months of age [5]. Several additional studies have also indicated performing total correction at any age between 3 and 11 months [6-8]. On the other hand, for patients in developing countries, establishing the diagnosis of TOF beyond adolescence is not uncommon [9]. The primary causes of the delayed diagnosis are patients’ late referrals because of incomplete information at the time of referral and treatment feasibility.

Treating TOF beyond infancy presents significant challenges, such as potential postoperative complications, for example, biventricular dysfunctions, arrhythmias, a postoperative gradient across the right ventricular outflow tract (RVOT), and pulmonary regurgitation [10], along with consequences of undetected TOF over time such as myocardial dysfunction and neurological disturbances [9]. Prolonged exposure to hypoxia in these patients may result in multiorgan failure [11-16]. Furthermore, the increasing enlargement of the right ventricle (RV) increases the risk of myocardial injury and arrhythmia [17].

Despite the well-established advantages of prompt and early surgery, individuals may be referred later in life, particularly in underdeveloped nations. A 10-year study found that the survival rate for individuals who were referred at an older age was 24% if surgery was not performed [4].

Due to the limited available literature on late total repair, we retrospectively analyzed different surgical techniques, perioperative patient recovery, and short- and long-term outcomes after late corrective surgery for TOF in children and adults [18].

Our study aims to provide a comprehensive understanding of postoperative complications, primarily RV dysfunction, during both the immediate postoperative period and follow-up. We also aim to evaluate the effect of transannular patch (TAP) or valve-sparing RVOT patch augmentation on hospital stays and non-ventriculotomy infundibular resection. This thorough approach ensures that our findings are reliable and can be applied in real-world medical settings.

## Materials and methods

This retrospective cohort study included patient data from the Department of Cardiac/Thoracic/Vascular Surgery, Vardhman Mahavir Medical College and Safdarjung Hospital, New Delhi. Inclusion criteria included (1) patients weighing 10 kg and above who underwent total correction for TOF between January 16, 2018, and January 15, 2024, with/without collateral circulation management; and (2) McGoon’s ratio of more than 1.3 and above. Exclusion criteria included (1) patients weighing below 10 kg; (2) patients with a McGoon’s ratio of less than 1.3; and (3) other pathologies including double outlet right ventricle (DORV), atrioventricular (AV) canal defects, single ventricle pathology, DORV with PS, and pulmonary atresia. As it is a retrospective study, patient consent was not needed.

Cohort selection

This study focused on patient outcomes, specifically those who underwent repair for simple TOF or pentalogy of Fallot. Any patients weighing 10 kg or above who underwent total correction for TOF, with management of collateral circulation, were also included in the study. A total correction was defined as VSD closure and infundibular resection with or without valve-sparing RVOT reconstruction or TAP. Few patients underwent neither of the above, and few patients who underwent neither of the above had trans-RA infundibular resection total correction. The data were studied between January 2018 and January 2024.

Data collection

The data collection process was meticulous, ensuring the reliability of the study. We included variables such as gender, weight, and age at repair, as well as the type of surgery, namely, non-ventriculotomy infundibular resection, RVOT augmentation, or TAP. In cases of ambiguity, operative notes were manually reviewed. Postoperative complications, especially RV dysfunction during the immediate postoperative period and follow-up, were also included.

Statistical analysis

Descriptive statistics were calculated for variables of interest and included means with standard deviations, medians with interquartile ranges (IQRs), or counts and percentages. Comparison between RVOT, TAP, and non-ventriculotomy infundibular groups was done using the Mann-Whitney U test. P-values <0.05 were considered significant.

## Results

**Figure 1:**
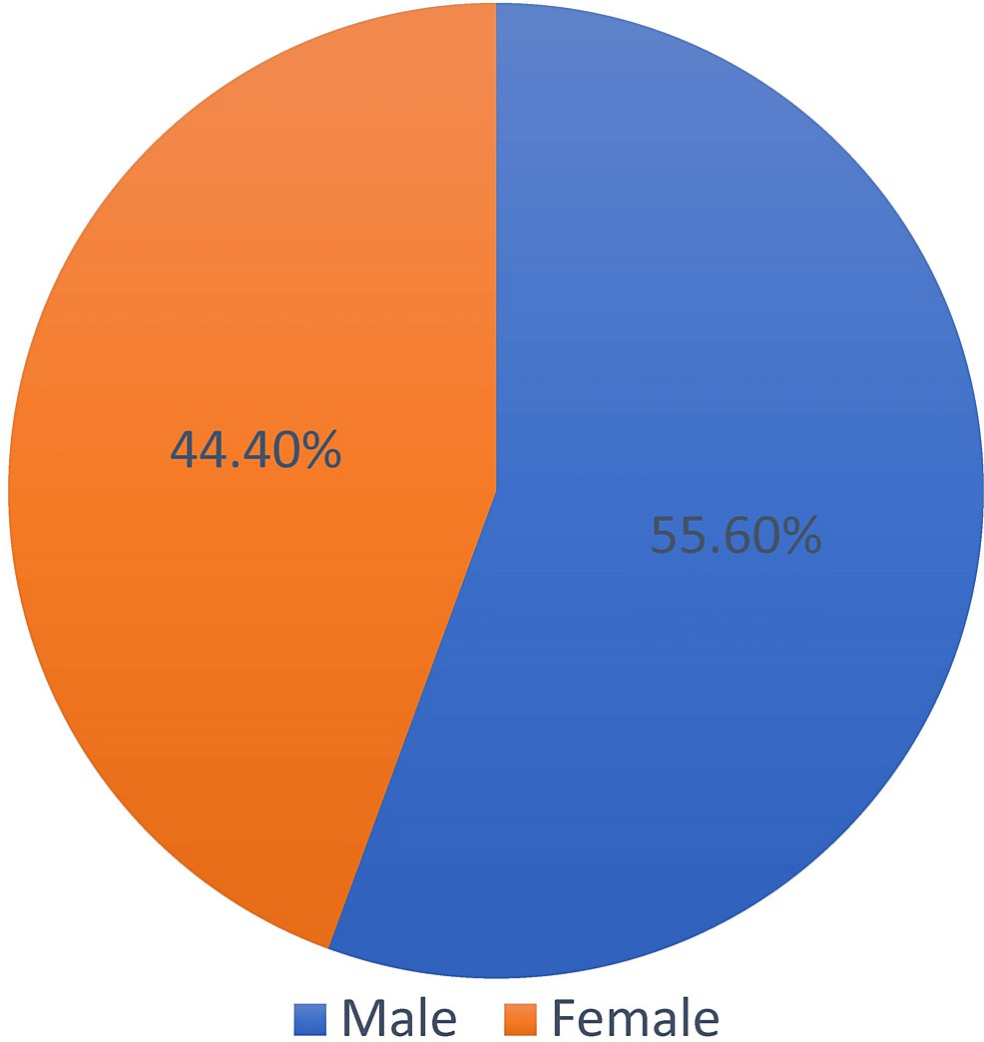
Pie chart of gender percentage.

**Figure 2:**
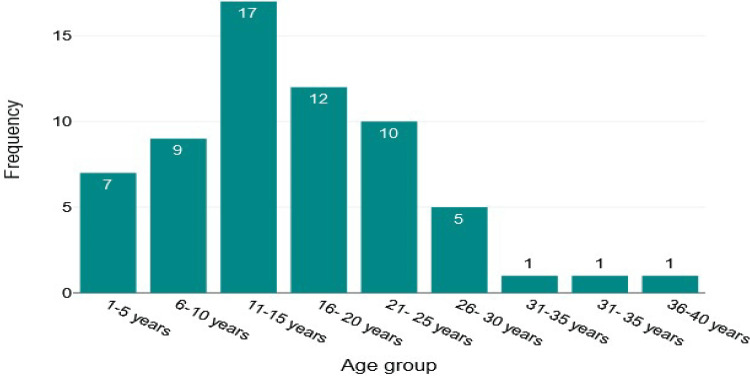
Histogram of age-wise distribution of patients.

**Figure 3:**
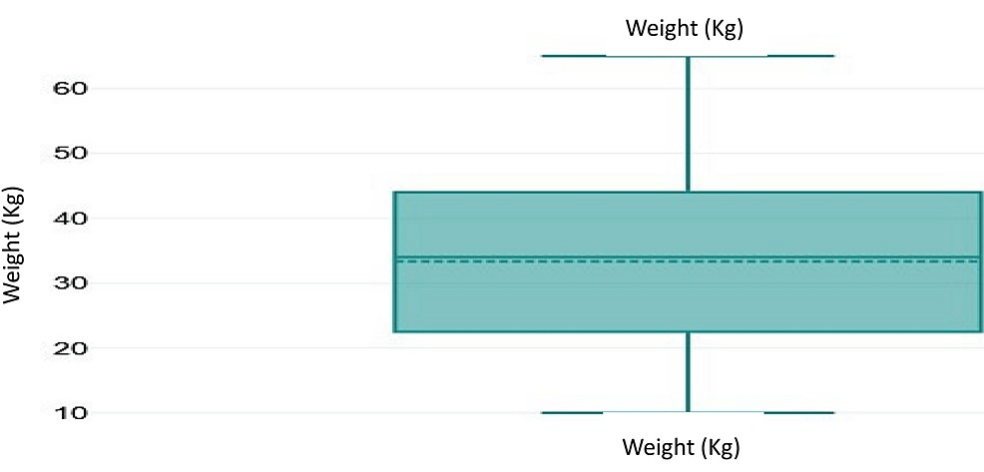
Weight distribution chart. Mean weight = 33.4 kg.

Among the study participants, 55.6% were males and 44.4% were females (Figure 1). Overall, 17 were 11-15 years old, 12 were 16-20 years old, 10 were 21-25 years old, nine were 6-10 years old, seven were 1-5 years old, and one each was 31-35 and 36-40 years old, with a mean age of 15.9 years (Figure 2). The mean weight of the study participants was 33.4 kg (Figure 3).

**Figure 4:**
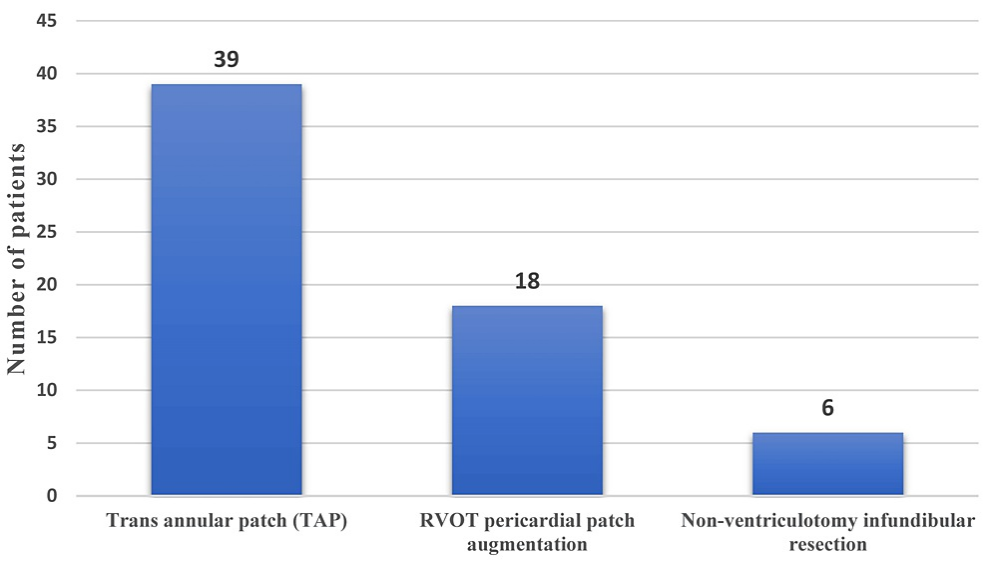
Type of surgical correction done. RVOT = right ventricular outflow tract

**Figure 5:**
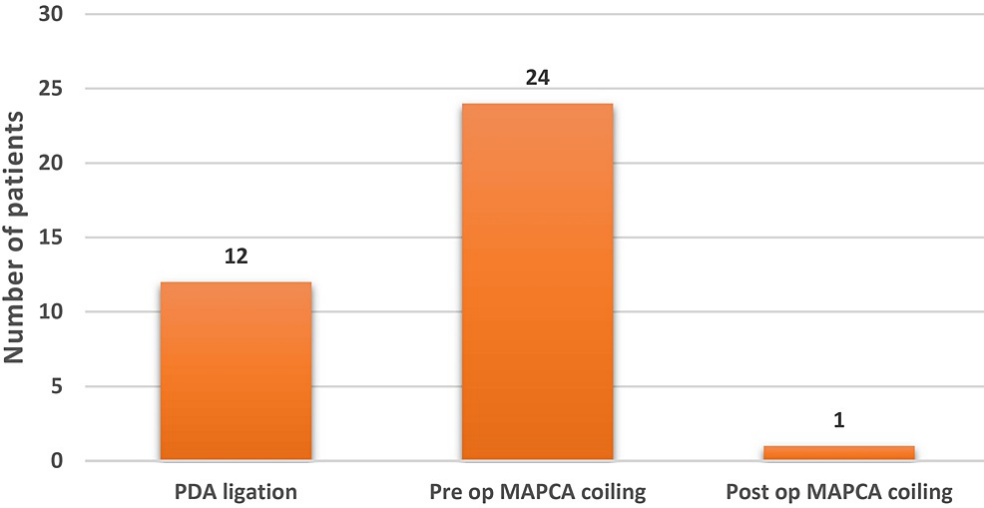
Collateral circulation management. PDA = patent ductus arteriosus; MAPCA = major aortopulmonary collateral arteries

Of the 63 patients, 39 underwent TAP surgery, 18 underwent RVOT pericardial patch augmentation, and six underwent non-ventriculotomy infundibular resection total correction (Figure 4). Major aortopulmonary collateral arteries (MAPCA) coiled in 25 patients (24 preoperatively and one postoperatively), and patent ductus arteriosus (PDA) ligation was done in 12 patients (Figure 5).

**Figure 6:**
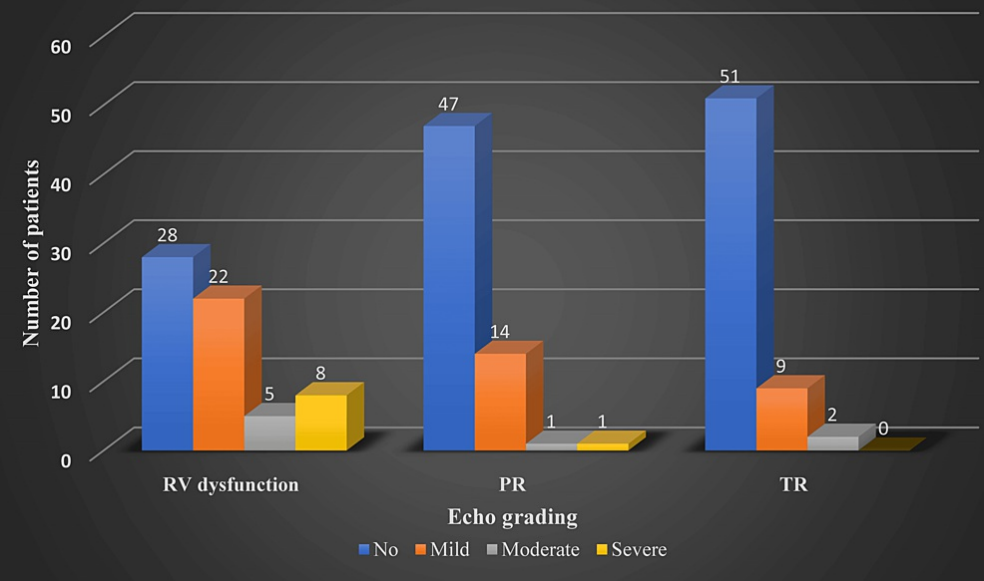
Immediate postoperative echocardiography reported complications. RV = right ventricular; PR = pulmonary valve regurgitation; TR = tricuspid valve regurgitation

**Figure 7:**
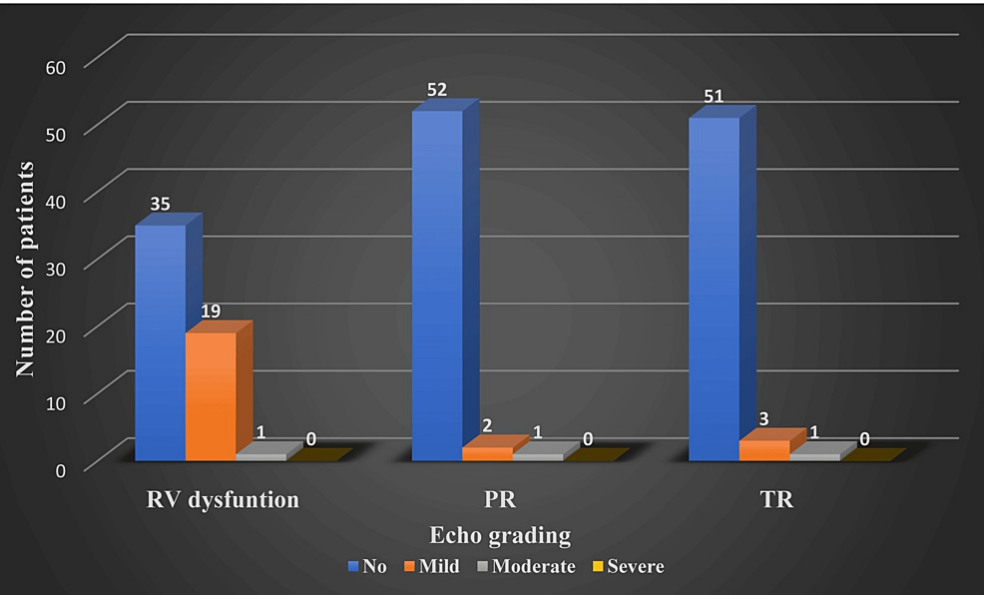
Follow-up echocardiography reported complications. RV = right ventricular; PR = pulmonary valve regurgitation; TR = tricuspid valve regurgitation

**Figure 8:**
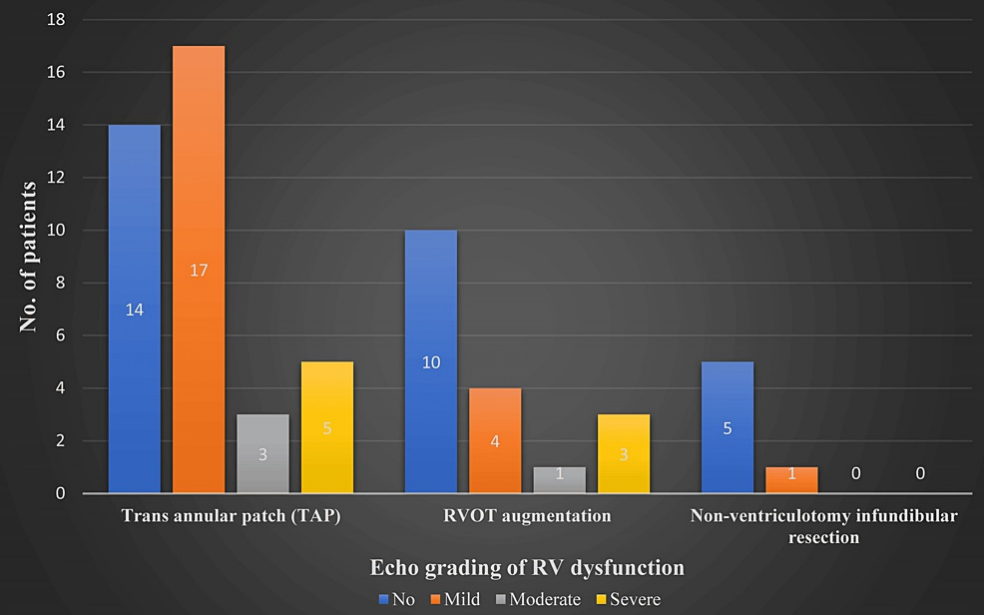
Grades of right ventricular dysfunction in postoperative echocardiography. TAP = transannular patch; RVOT augmentation = right ventricular outflow tract pericardial patch augmentation; RV = right ventricular

**Figure 9:**
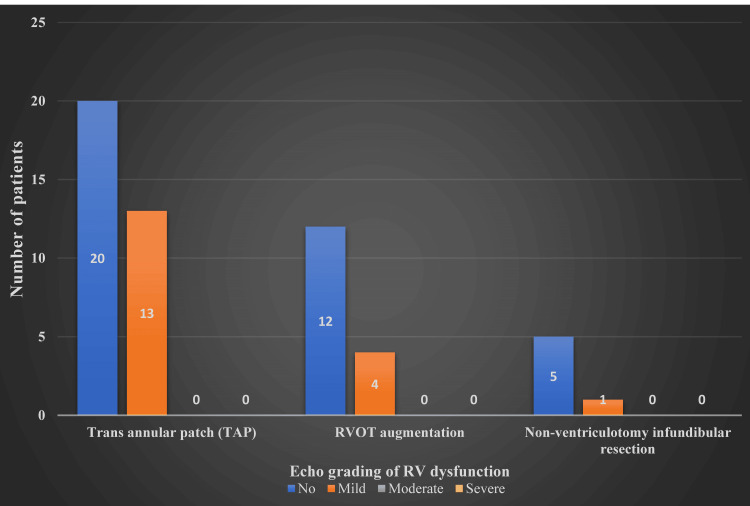
Grades of right ventricular dysfunction in follow-up echocardiography. TAP = transannular patch; RVOT augmentation = right ventricular outflow tract pericardial patch augmentation; RV = right ventricular

The Mann-Whitney U test results revealed a large effect size in the difference between age group and type of surgery without any significance in the weight class. Statistical significance was noted between patients undergoing a type of surgery and having varying degrees of RV dysfunction, more apparent among TAP patients (Figures 6-9). The difference was statistically significant at a 5% significance level. However, the significance is lost when assessing long-term follow-up.

## Discussion

The average lifespan of a patient with TOF who does not undergo surgical repair is 12 years. It has been estimated that only 11% survive to 21 years, 6% remain alive at 30 years, and 3% remain alive at 40 years [19]. Despite the proven success of total correction for TOF in less than one year, which was as high as 95% [18], our institutional exposure was limited. From January 2018 until January 2024, 119 total correction surgeries were done for TOF, of which 23 cases were pentalogy of Fallot. The institutional protocol was developed to accept TOF for total correction with McGoon’s ratio >1.5 with few exemptions not less than the ratio of 1.3 [20]. Over the period, the institutional practice was directed toward accepting patients weighing 10 kg and above [21] for total correction of TOF, considering amenable postoperative outcomes from cardiac and anesthetic recovery [22].

Another characteristic observation was a higher percentage of MAPCAs (40%) and PDAs (19%) compared to the international pediatric population (5-8%) [23], which is due to the physiological response to cyanosis and chronically reduced pulmonary blood flow. MAPCAs are notorious for pulmonary edema postoperatively, erosion and gross enlargement of bronchi, massive hemoptysis, and prolonged postoperative need for intensive care unit stay and mechanical ventilation [24]. To avoid such complications, MAPCAs were coiled with Cook coils transcatheterally by our cardiologists before surgical correction, and PDAs were suture ligated intraoperatively before proceeding to cardiopulmonary bypass after cannulations. Collateral vessels were successfully managed using this strategy. Thus, favorable postoperative outcomes were achieved except in one patient who was reported to have non-significant aortopulmonary circulation. Postoperatively, the patient required MAPCA coiling, but the patient eventually succumbed.

Surgical technique

Surgical techniques included median sternotomy and right vertical pericardiotomy preserving pericardium, if required, and standard aortic and bi-caval cannulation with looping cavae. Cardiopulmonary bypass was conducted in a state of mild hypothermia after heparinization and target ACT. The aorta was cross-clamped, and antegrade cardioplegia was administered. VSD closure was done using a transatrial approach or an RVOT incision in case of RVOT enlargement by patch plasty. A Hegar’s dilator was used to size the annulus appropriately for age after infundibular resection. The traditional practice of corrective surgery for TOF is to relieve RVOT stenosis and surgically close intracardiac shunting. For acceptable RVOT stenosis correction, surgeons universally perform various procedures such as pulmonary valvotomy, pulmonary artery angioplasty, and hypertrophied infundibular muscle resection to a possible extent, preserving the moderator band. Few cases did not require separate ventriculotomy for complete infundibular resection, while in some cases, an additional infundibular area right ventriculotomy to resect hypertrophied infundibular muscles was needed, thus preserving the pulmonary valve annulus. In many cases, the pulmonary valve annulus was sacrificed for transannular RVOT widening with Z-score <−3, warranting TAP for relieving PS. The transvalvular pressure gradient between RV and PA was routinely assessed following weaning from cardiopulmonary bypass, and the ratio of pressure across RV and left ventricle (LV) was <0.8 to establish adequate correction [25].

Postoperative course

Patients were shifted to the intensive care unit with ionotropic support. They were extubated on postoperative day (POD) one if uneventful and generally on POD two after postoperative echocardiography by the cardiologist. Patients were shifted to a high dependency unit (HDU) a day to two later. Subsequently, they were discharged. In our study, the postoperative average stay was 11 ± 4 days, which was comparable to the study by Benbrik et al., who compared patients from developed and developing for shorter durations of mechanical ventilation, intensive care unit stays, and hospital stays [26].

In 23 of 63 patients, a patent foramen ovale was created to decompress the right atrial, considering RV compliance reduction, leaving residual cyanosis [27]. During the closure of the VSD, care was taken to avoid injury to the bundle of His, thereby preventing AV conduction block. However, two patients (3%) developed complete AV nodal block requiring permanent pacemaker insertion.

There were eight in-hospital mortalities: one patient who had a high gradient across RVOT with residual 1-2 mm muscular VSD expired on POD one, two patients succumbed to intractable arrhythmia refractory to DC cardioversion after POD three, while five patients expired due to severe RV dysfunction causing low cardiac output syndrome within POD 10.

Of the remaining 55 who survived, two (3.1%) were re-explored for bleeding, and two (3.1%) were re-operated on POD one for residual VSD. Compared to the study by Khalid et al., 2.9% required re-exploration because of bleeding or tamponade, and 8.7% for residual VSD [9,28].

In the immediate postoperative period, as depicted in Figure 6 and Figure 8, all 63 patients underwent bedside transthoracic echocardiography, and their results are as follows: 56% had RV dysfunction based on tricuspid annular plane systolic excursion grading (n = 35: mild, 22; moderate, 5; severe, 8); 25% had pulmonary regurgitation (n = 16: mild, 14; moderate, 1; severe, 1); and 17% had tricuspid regurgitation (n = 11: mild, 9; moderate, 2).

As depicted in Figure 8, the degree of RV dysfunction was assessed in each of the three surgical techniques used. Regarding TAP, of the 39 patients, 25 had RV dysfunction. Seventeen had mild, three had moderate dysfunction, and five had severe RV dysfunction. Of the five patients who had severe dysfunction, four expired, and the only surviving patient with severe RV dysfunction was the lone post-modified Blalock-Taussig shunt patient who was part of our study and had a more extended hospital stay of more than two months and was discharged with moderate RV dysfunction.

Regarding RVOT pericardial patch augmentation, of 18 patients, eight had RV dysfunction. Overall, four had mild, one had moderate, and three had severe dysfunction. Of the three patients with severe dysfunction, two expired, and the only surviving patient had a sternum left open for 48 hours, which was later closed. The patient was discharged with mild RV dysfunction after a 10-day hospital stay. Regarding non-ventriculotomy infundibular resection, of six patients, only one had mild RV dysfunction.

The findings of the Mann-Whitney U test indicated that the difference between the type of operation and RV dysfunction had a significant impact, with the TAP having larger values. At a significance level of 5%, the difference was statistically significant.

Follow-up

Of the 55 discharged patients, follow-ups were categorized into short-term and long-term follow-ups based on recent outpatient visit dates from the surgery date. Short-term follow-ups were conducted for seven patients and 48 patients underwent long-term follow-ups. Of the 48 patients, seven had more than five years of follow-up, 16 had more than four years of follow-up, five had more than three years of follow-up, and 10 patients had follow-ups for more than two years and one year each.

During follow-ups, patients underwent clinical evaluation, including SpO_2_ measurement in room air and echocardiogram reports [27]. In the follow-up period, patients’ average peripheral saturation (SpO_2_) was 95% (91-100%), and the RV dysfunction had significantly regressed to a lower degree, as seen in Figure 7 and Figure 9. This suggests that irrespective of the type of surgical method employed, patients’ RV compliance improved with time [28-30].

Strengths and limitations

The study’s main advantage is an analysis of the outcomes of different surgical modalities and the long-term follow-up in the population of concern, thus aiding in successfully planning appropriate surgical management of TOF despite limited published articles. The main limitation is that this is a single-center study without a substantial sample size for generalizing the study findings.

## Conclusions

According to research, when TOF patients undergo total correction at an earlier age, their long-term survival is significantly better. Universally, the first year of life is the gold standard for the surgical management of patients with TOF. Short-term results of total correction for TOF in adults depend on cardiac anatomy and clinical status before surgery. Surgery may engender many sequelae and require prolonged medical attention. The outcome of surgery should not be measured by morbidity, mortality, and survival alone but also by the long-term quality of life after total correction in an otherwise average population but a special cohort for the disease in focus. Although TAP has significant immediate postoperative complications compared to other techniques, long-term follow-up suggests that long-term survival and quality of life, measured by major adverse cardiac events such as heart failure, arrhythmias, and reoperation rates, are commensurable in adulthood. This indicates that despite the initial challenges, TAP can provide satisfactory outcomes in the long run.
